# Liver kinase B-1 modulates the activity of dopamine neurons in the ventral tegmental area and regulates social memory formation

**DOI:** 10.3389/fnmol.2024.1289476

**Published:** 2024-04-05

**Authors:** Meng Yu, Fengjiao Sun, Guo Xiang, Yuhan Zhang, Xuejun Wang, Xia Liu, Bin Huang, Xingang Li, Di Zhang

**Affiliations:** ^1^Department of Neurosurgery, Qilu Hospital, Cheeloo College of Medicine and Institute of Brain and Brain-Inspired Science, Shandong University, Jinan, China; ^2^Jinan Microecological Biomedicine Shandong Laboratory and Shandong Key Laboratory of Brain Function Remodeling, Jinan, China; ^3^Institute of Metabolic and Neuropsychiatric Disorders, Binzhou Medical University Hospital, Binzhou, China

**Keywords:** Lkb1, social memory, dopamine neuron, ventral tegmental area, burst firing

## Abstract

Social memory is the ability to discriminate between familiar and unknown conspecifics. It is an important component of social cognition and is therefore essential for the establishment of social relationships. Although the neural circuit mechanisms underlying social memory encoding have been well investigated, little focus has been placed on the regulatory mechanisms of social memory processing. The dopaminergic system, originating from the midbrain ventral tegmental area (VTA), is a key modulator of cognitive function. This study aimed to illustrate its role in modulating social memory and explore the possible molecular mechanisms. Here, we show that the activation of VTA dopamine (DA) neurons is required for the formation, but not the retrieval, of social memory. Inhibition of VTA DA neurons before social interaction, but not 24 h after social interaction, significantly impaired social discrimination the following day. In addition, we showed that the activation of VTA DA neurons was regulated by the serine/threonine protein kinase liver kinase B1 (Lkb1). Deletion of Lkb1 in VTA DA neurons reduced the frequency of burst firing of dopaminergic neurons. Furthermore, Lkb1 plays an important role in regulating social behaviors. Both genetic and virus-mediated deletions of Lkb1 in the VTA of adult mice impaired social memory and subsequently attenuated social familiarization. Altogether, our results provide direct evidence linking social memory formation to the activation of VTA DA neurons in mice and illustrate the crucial role of Lkb1 in regulating VTA DA neuron function.

## Introduction

Impaired social interactions are frequently observed in patients with autism spectrum disorder (American Psychiatric Association, 2013). In addition to reduced attention to social stimuli, patients with autism spectrum disorder display impaired performance in tasks involving memory of both novel and familiar social stimuli (Dawson et al., 2002; Gomot et al., 2006; Kleinhans et al., 2009; Webb, 2009). Social memory refers to the ability to recognize and form memories of familiar social conspecifics as part of social cognition and is essential for the establishment of successful social interactions (McGraw and Young, 2010; Okuyama et al., 2016). The formation of social memory requires individuals to discriminate between novel and familiar sensory information and store it in long-term memory. However, the neural circuits and molecular mechanisms underlying the formation and regulation of social memory remain unclear.

Dopamine (DA) neurons in the ventral tegmental area (VTA) have been studied for their critical role in regulating behaviors related to motivation and reward processing (Schultz et al., 1993; Matsumoto and Hikosaka, 2009; Bamford et al., 2018; Saunders et al., 2018). Recent studies have revealed that DA neurons in the VTA are closely involved in the regulation of social behaviors. *In vivo* recordings in freely moving mice have revealed that DA neurons in the VTA are largely activated upon social interactions, and optogenetic activation of VTA dopamine neurons increases the exploration time for same-sex social targets through projections to the nucleus accumbens (Gunaydin et al., 2014). The DA neurons in the VTA show remarkable diversity (Morello and Partanen, 2015; Poulin et al., 2020). A subsequent study further demonstrated the role of DA neuron subpopulations in encoding specific modes of social contact and confirmed the recruitment of the VTA dopamine reward system in social reinforcement learning (Solie et al., 2022). Recruitment of the DA reward system facilitates memory formation in both associative and non-associative learning (Fadok et al., 2009; Ardiel et al., 2016; Nyberg et al., 2016; Frick et al., 2022). Recent studies have shown that social memory is retained in the hippocampus. Optogenetic stimulation of cells reactivated during re-exposure to familiar conspecifics in the ventral CA1 elicits the recall of social memory (Okuyama et al., 2016; Tao et al., 2022). Midbrain dopamine neurons project directly to the hippocampus (Gasbarri et al., 1994a), and dopamine release in the hippocampus is necessary for long-term potentiation and facilitates the persistence of long-term memories (Huang and Kandel, 1995; Otmakhova and Lisman, 1998). These findings raise questions regarding whether the dopamine reward system in the VTA is involved in the formation of social memory.

The activity of DA neurons in the VTA is modulated by both intrinsic and extrinsic factors. The serine/threonine protein kinase liver kinase B1 (Lkb1) was originally identified as a master regulator of the anterior–posterior axis of the Caenorhabditis elegans zygote (Kemphues et al., 1988). Accumulating evidence indicates that Lkb1 serves as a master upstream kinase that regulates various cellular processes, including cell polarization, metabolism, and growth (Lizcano et al., 2004; Shackelford and Shaw, 2009). Lkb1 is extensively expressed in various embryonic and adult tissues, including those of the central nervous system (CNS) (Jenne et al., 1998; Barnes et al., 2007). Although the function of Lkb1 in the peripheral tissue has been intensively studied, its role in the CNS is largely unexplored. Studies have revealed that Lkb1 is required for neural development and normal neural function. In the developing nervous system, Lkb1 is essential for key processes of axon formation, such as the establishment of axon/dendrite polarity (Barnes et al., 2007; Huang et al., 2014), axon initiation (Shelly et al., 2010), axon specification and axon growth (Courchet et al., 2013). In the mature nervous system, Lkb1 signaling in the hypothalamus has been reported to regulate glucose homeostasis in peripheral tissues through the secretion of a melanocyte-stimulating hormone (Claret et al., 2011). Moreover, Lkb1 acts as a key modulator of presynaptic neurotransmitter release by fine-tuning presynaptic Ca^2+^ homeostasis in cortical neurons (Billups and Forsythe, 2002; Kwon et al., 2016). Collectively, these results illustrate the important role of Lkb1 in the direct regulation of CNS development and function.

In this study, we illustrated the crucial role of VTA DA neurons in the regulation of social memory formation and identified Lkb1 as a key modulator of the activity of VTA DA neurons. The results demonstrated that intact VTA DA neuron excitability was required for the formation of social memory in familiar conspecifics but not in familiar objects. In addition, using conditional DA neuron knockout mice, we showed that Lkb1 expression in DA neurons was pivotal for the formation of social memory. Finally, Lkb1 was essential for maintaining the burst-firing activity of VTA DA neurons, as was the electrophysiological mechanism underlying its role in modulating social memory formation. In general, these findings support a novel role for VTA DA neurons in modulating social interactions and suggest that Lkb1 can be a molecular substrate for social learning.

## Materials and methods

### Animals

Male C57BL/6J wild-type mice were purchased from Beijing Vital River Laboratory Animal Technology Co (Beijing, China). Lkb1^flox/flox^ mice (Nakada et al., 2010) were kindly provided by professor Wencheng Zhang (Zhang et al., 2021). Lkb1^flox/flox^ mice were crossed with mice expressing the Cre recombinase under the control of the dopamine transporter (DAT-IRES-Cre) (Backman et al., 2006) (Stock# 006660, Jackson Laboratory, Bar Harbor, ME, USA) to generate Lkb1^flox/flox^ DAT-Cre mice. Male DAT-Cre^–/+^-Lkb1^flox/flox^ mice were crossed with female Lkb1^flox/flox^ mice to yield DAT-Cre^(+/–)^-Lkb1^flox/flox^ (termed Lkb1-DAT-Cre) mice and DAT-Cre^(–/–)^-Lkb1^flox/flox^ littermate controls (CTLs). All experiments were carried out using adult male mice (10– 12 weeks) except for the mice (6–7 week-old juveniles) used as social stimuli. The mice were kept on a 12-h light/dark cycle (lights on at 7 a.m.) with *ad libitum* access to food and water.

All experimental procedures were approved by the Institutional Animal Care and Use Committee of Qilu Hospital at Shandong University. All applicable international, national, and/or institutional guidelines for the care and use of animals were followed.

### Drugs

Clozapine N-oxide (CNO) was purchased from Sigma-Aldrich (Sigma-Aldrich Shanghai Trading Co, China) and dissolved in sterile saline (Sal).

### Stereotaxic surgeries

Stereotaxic surgery was performed as previously reported (Zhang et al., 2017; Xiang et al., 2023). Briefly, mice were anesthetized with isoflurane (3% induction and 1% maintenance) and mounted on a stereotaxic frame. For viral injection, a total volume of 1 μL AAV-hsyn-Cre-EGFP (OBiO, Shanghai, China) or adeno-associated virus containing a double-floxed inverted open reading frame expressing the engineered Gi-coupled receptor hM4D tagged with the fluorescent protein mCherry (AAV-DIO-hm4d-mCherry, OBiO) was bilaterally injected into the ventral tegmental area (AP: −3.1 mm, ML: ± 0.5 mm, DV: −4.5 mm from bregma) of Lkb1^flox/flox^ or DAT-IRES-Cre mice, respectively, using a 30-gauge stainless steel injector connected to a LEGATO 130 NL single-channel syringe pump (KD Scientific, Holliston, MA, US) at a rate of 0.125 μL/min. The injector was kept in the microinjection region for an additional 5 min to prevent backflow from injector withdrawal. The mice were allowed to rest for at least 3 weeks to ensure viral expression.

### Behavioral test

#### Social interaction tests

Social approach test: This test was conducted according to previous studies with slight modifications (Berton et al., 2006). The test was performed in an open plastic field (40 cm × 40 cm × 30 cm). The test mouse was introduced to the test arena and tracked for two 2.5-min sessions. During the first session, test mice were exposed to an empty wired cage located at the end of the chamber. During the second session, a social conspecific (a sex-matched juvenile mouse, 4–6 weeks old) was placed under a wired cage. The test mouse was removed from the arena between sessions. Social approaches were sampled and analyzed using ANY-maze video-tracking software (Stoelting Co., Wood Dale, IL, USA). Chambers were cleaned with 70% ethanol between mice.

Social memory test: This test was modified from the three-chamber test (Liu et al., 2022). Briefly, the mice were allowed to freely explore the apparatus (60 cm × 45 cm × 25 cm) made of white acrylic and divided into three interconnected compartments with open doors for three 10-min test trials. During the habituation trial, the test mouse was allowed to explore the test apparatus freely. In the social interaction trial, the test mouse was allowed to interact with either a sex-matched juvenile mouse (4–6 weeks old, S1) or an empty wired cage (pencil holder) placed upside down. In the social discrimination trial, a novel social conspecific (S2) was placed under an empty pencil holder, and the test mouse was free to choose between S1 and S2. A 24-h time interval was added between trials according to the experimental design. The locations of the empty pencil holder and social conspecifics were counterbalanced for each test mouse. All behaviors were recorded, and social approaches were analyzed using the ANY-maze software (Stoelting Co.). Chambers and enclosures were wiped with 70% ethanol between mice.

Social familiarization test: The test was performed as previously reported with slight modifications (Bariselli et al., 2018). A white acrylic arena (30 cm × 20 cm × 20 cm) with a size similar to that of a home cage was used for this test. The test consisted of a 3-day familiarization phase and a 1-day novelty phase. During the familiarization phase (3 days), the experimental mice were left free to explore the arena and interact with the same social conspecific (juvenile mouse, 4–6 weeks old, S1) held in a wired cage for 10 min daily. The experimental and stimulus mice were returned to their home cages after social interactions. On day 4, a novel social conspecific (S2) was introduced to the experimental mice for 10 min. The non-aggressive behaviors that the experimental mice initiated toward the social conspecifics were scored by an experimenter blinded to the genotype. The object familiarization test was performed as described above, using Lego blocks and small toys as objects.

Home-cage social interaction test: This test was performed in the new home cage of the experimental mice. Mice were habituated to the new home cage for 2 h before being introduced to a novel social conspecific (juvenile mouse, 4–6 weeks old) and were left free to interact for 10 min. The total amount of time that the experimental mice initiated and spent interacting with the social stimulus was quantified by experimenters blinded to the genotype. After a 2-h time interval, the mice were tested with Lego blocks as objects using procedures similar to those described above.

Mice were routinely handled by the experimenters for at least 3 days before initiation of any behavioral test batteries. For social behavioral tests, mice were left in the home cage for at least 1 week between different tests and a completely different object or juvenile mouse was used if performing the repeated tests to the same batch of mice to minimize confounding factors caused by repeated measures.

#### Buried food test

The buried food test was performed as previously described (Lehmkuhl et al., 2014) with minor modifications. Mice were food-deprived for 24 h prior to the test trials. On the test day, mice were tested in two trials. The mice were individually habituated to their new home cages filled with 8 cm of bedding material for 2 h before the test trials. During the first trial, the mouse was removed from the cage, and a food pellet size of 0.78 cm 2 cm × 2 cm (cylinder-shaped), which was provided daily, was buried 3 cm below the surface before the mouse was returned to the cage. During the second trial, the food pellets were replaced with roasted peanuts at a time interval of 1 h. The time spent retrieving food pellets or roasted peanuts was recorded and analyzed.

#### Novel object recognition test

The mice were placed in an apparatus made of white acrylic (40 cm × 40 cm × 20 cm) and allowed to explore freely for 10 min. Two identical cuboid objects (5 cm × 3 cm × 3 cm Lego blocks) were placed 8 cm from the corner of the apparatus. The mice were placed in the center of the arena and allowed to explore the objects for 10 min before returning to their home cages. The next day, one of the Lego blocks was replaced with a plastic cylindrical bottle filled with water, and the mice were placed back in the arena for 10 min. Objects were counterbalanced across subjects, and object positions were counterbalanced across trials. The total time spent exploring the object was quantified using the video-tracking software Limelight (Coulbourn Instruments, Whitehall, PA, USA).

#### Open field test

The open field test was performed as previously described (Xiang et al., 2023). The apparatus was made of white acrylic (40 cm × 40 cm × 20 cm) and divided into nine squares. The central square was defined as the central zone. The mice were placed in the center of the apparatus and allowed to explore for 10 min. The locomotion of the mice and the time spent in the center zone during the first 5 min were analyzed using the Limelight software (Coulbourn Instruments).

#### Elevated plus maze test

The elevated plus maze was purchased from RWD (Shenzhen, China). The maze comprised of two closed arms (30 cm × 5 cm × 15 cm) and two open arms (30 cm × 5 cm) placed 70 cm above the ground. The mice were placed in the intersection area with their heads facing the corner and allowed to freely explore the maze for 5 min. Exploratory activity was recorded and analyzed using ANY-maze software (Stoelting Co.).

### *In vivo* electrophysiology

*In vivo* electrophysiological recordings were performed as previously reported (Sun et al., 2019). Briefly, mice were anesthetized with 4% chloral hydrate (400 mg/kg, intraperitoneally) and placed in a stereotaxic apparatus with a heating pad to maintain the body temperature at 37°C. Anesthesia was maintained by the supplementary administration of chloral hydrate to suppress the limb compression withdrawal reflex. Extracellular recording electrodes were pulled from borosilicate glass capillaries and filled with 2 M NaCl containing 2% Chicago sky blue dye (impedance 6–14 MΩ). Electrodes were lowered into the VTA (coordinates: AP −3.1 mm, ML +0.5 mm, DV −4.5 mm from bregma) using a hydraulic microdrive, and 6–12 vertical passes were made throughout the VTA, separated by 100 μm. Spontaneously active cells were identified as putative dopamine neurons using previously defined electrophysiological parameters recorded with open-filter settings (low pass: 30 Hz; high pass: 30 kHz) (Grace and Bunney, 1983; Ungless and Grace, 2012; Sun et al., 2019). The average spontaneous firing rate (Hz) and average percent burst firing, defined as the occurrence of two consecutive spikes with an inter-spike interval >160 ms over 3 min were measured for each mouse.

### Immunohistochemistry

The mice were anesthetized with an intraperitoneal injection of avertin (250 mg/kg, Sigma-Aldrich, Shanghai, China) and transcardially perfused with 0.1 M phosphate-buffered saline (pH 7.4), followed by incubation in 4% paraformaldehyde. The brains were removed and post-fixed in 4% paraformaldehyde at 4°C overnight, followed by dehydration in a 30% sucrose solution. Brains were then sectioned into 40 μm coronal sections on a cryostat and stored in a cryoprotectant solution. For immunofluorescent staining, the sections were blocked in blocking buffer (1% BSA, 3% donkey serum, and 0.3% Triton X-100 in phosphate-buffered saline) for 1 h at room temperature and incubated with mouse anti-tyrosine hydroxylase (TH) primary antibody (1:400, #MAB318, Millipore, Temecula, CA, USA) and rabbit anti-c-fos primary antibody (1:400, #2250, Cell Signaling Technology, Danvers, MA, USA) overnight at 4°C. The next day, the brain sections were incubated with Alexa Fluor^®^ 488 goat anti-mouse IgG antibody (1:400, A-21202, Invitrogen, Carlsbad, CA, USA) and Alexa Fluor^®^ 594 goat anti-rabbit IgG antibody (1:400, A-21207, Invitrogen, Carlsbad, CA, USA) for 4 h. After washing with phosphate-buffered saline, the sections were mounted on coverslips with the ProLong Gold antifade reagent. The co-localization of TH with c-fos was observed using a Leica SP8 confocal microscope (Leica Microsystems, Wetzlar, Germany). Images of immunohistochemistry counting were acquired using confocal microscopy (Leica SP8) under a 10X objective. Neurons stained against c-Fos (red), mCherry (red), TH (red or blue), Cre (green) and DAPI (blue) were manually analyzed using ImageJ.

### Statistical analysis

Data are presented as the mean ± standard error of the mean. Statistical significance was assessed using a two-way analysis of variance with repeated measures or a two-tailed Student’s *t*-test (unpaired), where appropriate. Sidak’s *post-hoc* test was used to determine the significance between groups using analysis of variance. All statistical analyses were performed using GraphPad Prism 8.0 software (GraphPad Software, La Jolla, CA, USA). The results were considered significantly different at *p* < 0.05.

## Results

### VTA DA neurons are activated during social memory formation

**FIGURE 1:**
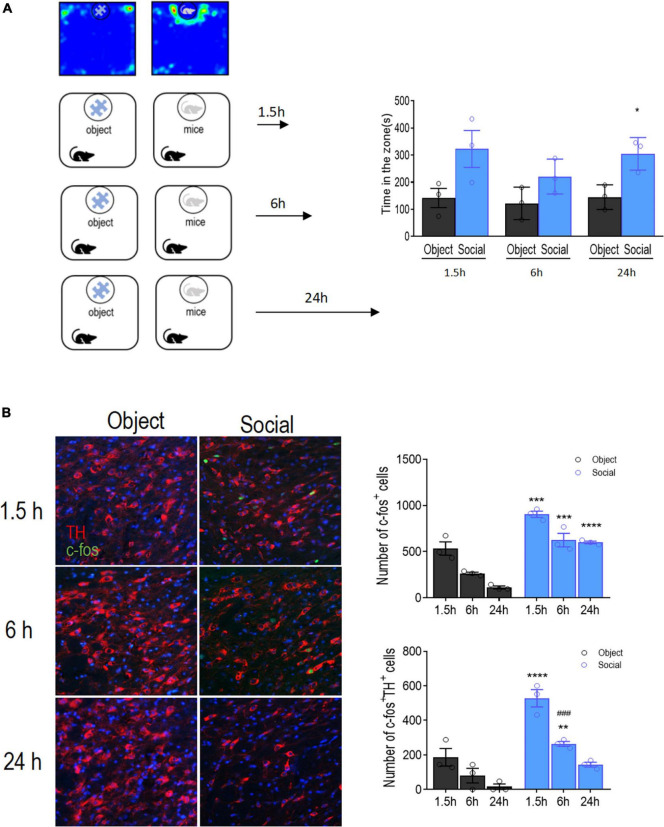
Activation time course of VTA DA neurons after social approach test. **(A)** Left: Schematic diagram of the social approach test. Two groups of mice (object vs. social) were allowed to freely interact with either a social conspecific or an object, respectively, for 10 min followed by transcardiac perfusion at different time points. Right: Mice in the social group spent significantly more time interacting with the target compared with that of mice in the object group. **(B)** Left: Representative images showing the activation of DA neurons in the VTA by co-immunofluorescent staining with Tyrosine hydroxylase (TH, red) and c-fos (green). Bar: 25 μm. Right: Quantification of the total number of activated cells (c-fos^+^), activated DA neurons (TH^+^ c-fos^+^), and the percentage of activated DA neurons. Each group contained 12 sections from 3 mice for all six groups. *p < 0.05, **p < 0.01, ***p < 0.001, ****p < 0.0001 compared with object; ^###^*p* < .0.001 compared with 1.5 h. Data are presented with mean ± S.E.M.

To determine whether the VTA dopamine system is involved in social memory processes, we tested the activation of VTA DA neurons during the formation and retrieval of social memories. Mice were sacrificed 1.5, 6, or 24 h after the social interaction test for immunofluorescence staining of TH and c-fos. We found that mice spent a significantly longer time interacting with the social conspecific than with the toy [target: *F*_(1, 4)_ = 44.81, *p* = 0.0026; time: *F*_(2, 8)_ = 0.9899, *p* = 0.4129; target × time: *F*_(2, 8)_ = 0.4079, *p* = 0.6781, Figure 1A]. Correspondingly, when probed at 1.5 h, the number of positive cells stained for c-fos in the VTA was significantly higher in mice interacting with a social conspecific than in mice exploring the toy [targets: *F*_(1, 4)_ = 151.6, *p* = 0.0003; time: *F*_(2, 8)_ = 31.41, *p* = 0.0002; targets × time: *F*_(2, 8)_ = 1.107, *p* = 0.3764].

Double immunofluorescence staining showed that the total number of cells positive for TH and c-fos was significantly increased in the VTA of mice interacting with social conspecifics [targets: *F*_(1, 4)_ = 32.15, *p* = 0.0048; time: *F*_(2, 8)_ = 51.65, *p* < 0.0001; targets × time: *F*_(2, 8)_ = 7.916, *p* = 0.0127; Figure 1B], indicating that the DA neurons in the VTA were activated upon social interaction. Memory consolidation occurs within 6 h of learning (Okuda et al., 2021). Hence, we further examined the activation of VTA DA neurons 6 h after social interaction. The results showed that the number of c-fos- and TH-positive cells was reduced at 6 h compared with that at 1.5 h in mice interacting with the social conspecifics (*p* = 0.0005), indicating a gradual decline of DA neuron activity. Despite this decline, there were still significantly more c-fos- and TH-positive cells in the VTA in mice interacting with social conspecifics than in mice interacting with the toy (*p* = 0.0095), suggesting residual activation of VTA DA neurons at 6 h after social interaction. When compared at 24 h, we only observed a significantly higher number of c-fos-positive cells, while the number of double-positive cells in the VTA was not significantly different between the social and object groups. The sustained increase in the number of c-fos expressing VTA DA neurons after social interaction suggests that VTA DA neurons participate in regulating social memory consolidation.

### Inhibition of VTA DA neurons blocks the formation of social memory

**FIGURE 2:**
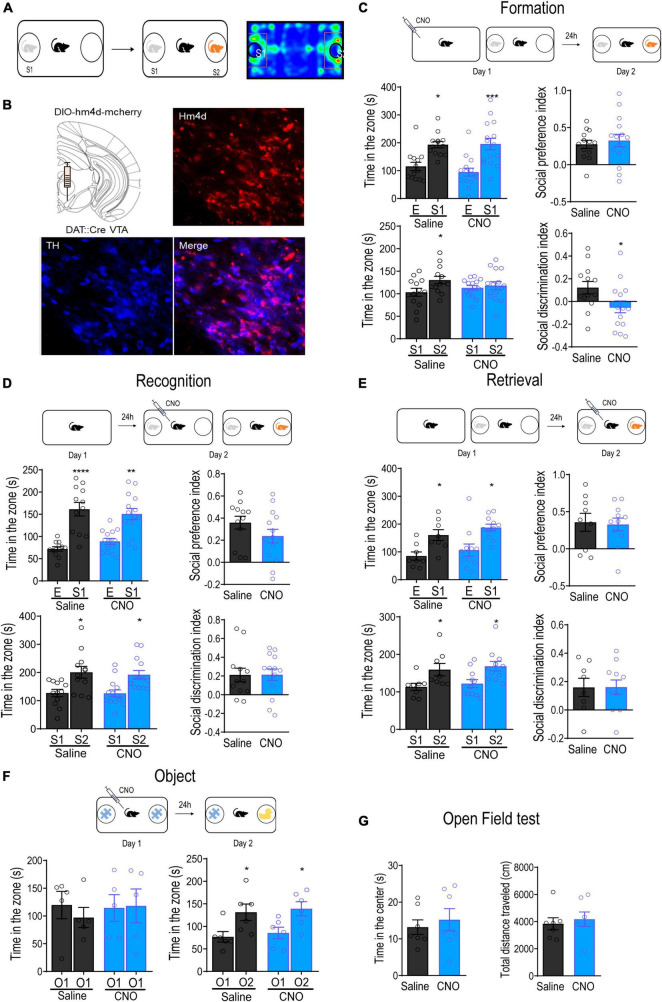
Inhibition of VTA DA neurons attenuated the formation of social memory. **(A)** Schematic diagram of the social memory test. **(B)** Representative immunofluorescence images of dopamine transporter (DAT)-Cre transgenic mice with the injection of AAV-DIO-hM4D-mCherry into the VTA. Blue: TH^+^; Red: hM4D. Bar: 25 μm. **(C)** Inhibition of VTA DA neurons before the formation of social memory. Injection of CNO (1 mg/kg) 30 min before introducing the test mouse to the social conspecific 1 (S1) impaired discrimination between S1 and novel social conspecific (S2) tested 24 h later. *p < 0.05, ***p < 0.001 compared with E, S1 or saline. **(D)** Inhibition of VTA DA neurons before social discrimination. Injection of CNO (1 mg/kg) 30 min before introducing the test mouse to S1 did not affect the social discrimination between S1 and S2 tested immediately. *p < 0.05, **p < 0.01, ****p < 0.0001 compared with E, S1 or saline. **(E)** Inhibition of VTA DA neurons before the retrieval of social memory. Injection of CNO (1 mg/kg) 24 h after introducing the test mouse to S1 did not affect the discrimination between S1 and S2. *p < 0.05 compared with E, S1 or saline. **(F)** Inhibition of VTA DA neurons before object recognition test. Injection of CNO (1 mg/kg) before introducing the test mouse to the Lego block (O1) did not affect object discrimination between O1 and a water bottle (O2). *p < 0.05 compared with O1. **(G)** Open field test. Inhibition of VTA DA neurons did not alter either the time spent in the center or the total distance traveled in mice. Data are presented with mean ± S.E.M.

Given that VTA DA neurons are activated during social interactions, we further investigated the role of VTA DA neurons in regulating social memory processes using a modified three-chamber test. Mice were allowed to freely explore a three-chambered arena for 10 min during the habituation session, followed by a 10-min social interaction session, during which they were allowed to interact with either an empty wired cage or a social conspecific (S1) in a wired cage. Social discrimination was tested at different time intervals after the social interaction sessions. During the social discrimination session, the experimental mice were given choices to interact with a familiar social conspecific (S1) or a novel social conspecific (S2) (Figure 2A). To specifically inhibit VTA DA neurons, we microinjected an AAV-DIO-hm4d-mCherry into the VTA of DAT- Cre transgenic mice. Immunofluorescent staining for TH revealed positive hm4d expression in 82.48 ± 4.2% of VTA DA neurons (Figure 2B). Limited mCherry expression was observed in the SNc and we did not observe any virus-mediated expression in SNr or red nucleus.

To test whether the activation of VTA DA neurons was required for the formation of social memory, we selectively inhibited VTA DA neurons using a single injection of CNO (i.p., 1 mg/kg) 30 min before the initial social interaction. Our results showed that mice injected with either Sal or CNO showed a clear preference for social conspecifics over the empty wired cage during the initial social interaction [group: *F*_(1,26)_ = 0.4256, *p* = 0.5199; target: *F*_(1,26)_ = 24.81, *p* < 0.0001; group × target: *F*_(1,26)_ = 0.4081, *p* = 0.5285; Figure 2C], leading to a similar social preference index [calculated as (tS1-tE)/(tS1+tE)] between the groups [t(26) = 0.4942, *p* = 0.6253]. When tested for discrimination between familiar and novel social conspecifics the next day, mice in the Sal group spent significantly more time interacting with the novel social conspecific S2 [group: *F*_(1,26)_ = 0.0184, *p* = 0.8930; target: *F*_(1,26)_ = 4.702, *p* = 0.0395; group × target: *F*_(1,26)_ = 2.222, *p* = 0.1481], indicating the successful formation of memory of the familiar social conspecific S1. However, the mice injected with CNO spent a similar amount of time interacting with S1 and S2 [t(28) = 0.4949, *p* = 0.6245], exhibiting an impairment in social discrimination. Indeed, the social discrimination index, calculated as (tS2-tS1)/(tS2+tS1), was significantly reduced in mice injected with CNO [t(26) = 2.217, *p* = 0.0356].

The failure of social memory formation may have been due to the inability to discriminate between the sensory information of different social conspecifics. To rule out this possibility, we injected Sal or CNO 30 min before the initial social interaction and immediately tested for discrimination. The results showed that mice in both groups showed similar preferences for the social conspecific [group: *F*_(1,24)_ = 0.1046, *p* = 0.7492; target: *F*_(1,24)_ = 42.08, *p* < 0.0001; group × target: *F*_(1,24)_ = 1.438, *p* = 0.2421], as well as for the novel social conspecific S2 [group: *F*_(1,24)_ = 0.1237, *p* = 0.7281; target: *F*_(1,24)_ = 17.77, *p* = 0.0003; group × target: *F*_(1,24)_ = 0.04565, *p* = 0.8326; Figure 2D]. Correspondingly, the social preference index and social discrimination index were also not significantly different between the groups [social preference index: t(24) = 1.422, *p* = 0.1678; social discrimination index: t(24) = 0.02951, *p* = 0.9769], indicating normal discrimination between different social conspecifics in CNO-injected mice. Novelty experiences induce the activation of DA neurons in the VTA (Duszkiewicz et al., 2019). To rule out the possibility that the inhibition of VTA DA neurons impairs social discrimination by dampening novelty-induced memory consolidation rather than blocking social memory formation itself, mice were subjected to novel object recognition tests with a 24-h time interval. As shown in Figure 2F, inhibition of VTA DA neurons with an object during initial exploration did not affect discrimination between familiar and novel objects 24 h later [group: *F*_(1,10)_ = 0.2822, *p* = 0.6069; target: *F*_(1,10)_ = 14.68, *p* = 0.0033; group × target: *F*_(1,10)_ = 0.0008, *p* = 0.9779, right panel], suggesting that inhibition of VTA DA neurons did not affect novelty enhancement of object memory. Collectively, these results indicate that the activation of VTA DA neurons during initial social interaction is essential for the formation of social memory.

To confirm that DA neurons are required for the formation of social memory, we examined whether the VTA dopamine system is also involved in the retrieval of social memory. A different batch of mice were used in this test. At 24 h after the initial social interaction, mice were administered a single injection of either Sal or CNO before being subjected to the social discrimination test. Mice in both groups preferred to interact with S2 instead of S1 [group: *F*_(1,17)_ = 0.5116, *p* = 0.4842; target: *F*_(1,17)_ = 12.49, *p* = 0.0025; group × target: *F*_(1,17)_ = 0.0002, *p* = 0.9864], and the social novelty preference index was not significantly different between groups [t(17) = 0.01741, *p* = 0.9863]. Given that all mice spent a similar amount of time interacting with the S1 during the initial social interaction, these results indicated that social memory retrieval did not recruit VTA DA neurons (Figure 2E). Collectively, these results reveal that the activation of VTA DA neurons is required for social memory formation instead of social memory retrieval. Finally, locomotor activity was not affected by the inhibition of VTA DA neurons, as observed in the open-field test [time: t(12) = 0.5600, *p* = 0.5858; distance: t(12) = 0.4854, *p* = 0.6361; Figure 2G].

### Lkb1 deficiency reduces the burst-firing activities of VTA DA neurons

**FIGURE 3:**
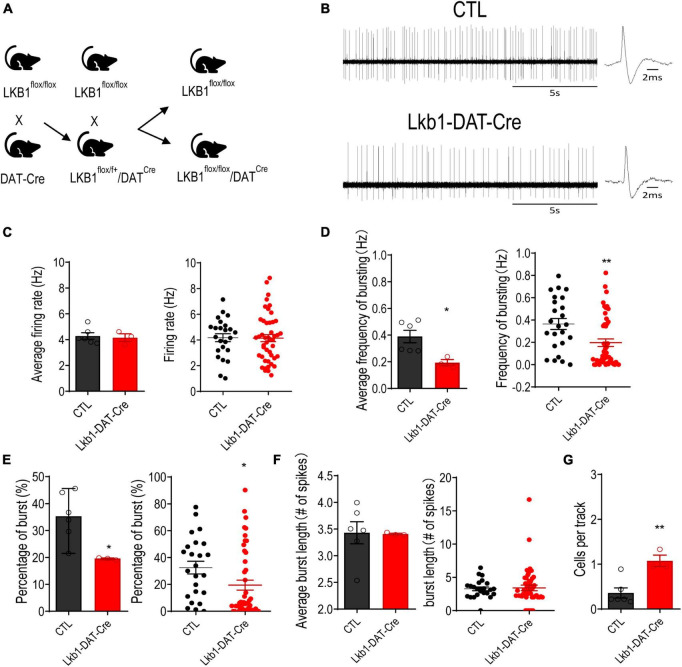
Liver kinase B1 (Lkb1) deficiency reduces the burst-firing activities of VTA DA neurons. **(A)** Lkb1-DAT-Cre mice were generated by breeding the Lkb1^flox/flox^ mice and mice expressing Cre recombinase under the control of the dopamine transporter (DAT-IRES-Cre) to generate Lkb1flox/flox, DAT-Cre mice. The male DAT-Cre^–/+^-Lkb1^flox/flox^ mice were crossed to female Lkb1^flox/flox^ mice, to generate DAT-Cre^+/–^-Lkb1^flox/flox^ (termed Lkb1-DAT-Cre) mice and DAT-Cre^–/–^-Lkb1^flox/flox^ littermate controls (termed CTL). **(B)** Representative *in vivo* VTA DA neuron firing traces from CTL and Lkb1-DAT-Cre mice. **(C)** Average firing rate and scatter plot distribution of individual data points. (Lkb1-DAT-Cre: *n* = 46 from 3 mice, CTL: *n* = 24 from 6 mice). **(D)** Average burst firing frequency and scatter plot distribution of individual data points. **(E)** Average spikes within burst and scatter plot distribution of individual data points. **(F)** Average burst length and scatter plot distribution of individual data points. **(G)** Populational activity of VTA dopamine neurons. *p < 0.05, **p < 0.01 compared with CTL. Data are presented with mean ± S.E.M.

DA neuronal activity is regulated by both intrinsic and extrinsic factors (Grace and Bunney, 1983). Lkb1, a master regulatory kinase related to various important cellular processes such as cell metabolism, cell proliferation, and cell polarity is ubiquitously expressed in all fetal and adult tissues. It regulates the development and activity of several types of neurons (Baas et al., 2004; Forcet et al., 2005; Radu et al., 2019). Western blotting confirmed that Lkb1 was expressed in the VTA (Supplementary Figure 1). The immunofluorescence of Cre expression was selective for dopaminergic neurons, suggesting that Lkb1 loss would be confined to only those cells in Lkb1-DAT-Cre mice (Supplementary Figure 2A). We examined whether Lkb1 played a role in regulating the firing properties of DA neurons. Lkb1^fl/fl^ mice with exons 3–6 flanked by two LoxP sites (Nakada et al., 2010) were crossbred with DAT-IRES-cre mice to generate Lkb1-DAT-Cre mice (Figure 3A). The *in vivo* firing properties of the putative VTA DA neurons were examined using an extracellular single-unit recording technique (Figure 3B). Our results showed no difference in the tonic firing rate between Lkb1-DAT-Cre mice and littermate CTLs [average: t(7) = 0.3024, *p* = 0.7711; cell: t(68) = 0.07425, *p* = 0.9410; Figure 3C] when averaged to the number of mice (left panel) or compared between the cells recorded from the two groups (right panel). However, when analyzed for phasic/burst activity, the frequency of bursting was significantly lower in Lkb1-DAT-Cre mice than in littermate CTLs [average: t(7) = 2.831, *p* = 0.0254 (left panel); cell: t(68) = 2.846, *p* = 0.0058 (right panel); Figure 3D]. The decrease in the frequency of bursting was accompanied by a decrease in the percentage of bursting [average: t(7) = 2.914, *p* = 0.0225 (left panel); cell: t(68) = 2.122, *p* = 0.0375 (right panel); Figure 3E]. In contrast, burst length was similar between the two genotypes [average: t(7) = 0.07877, *p* = 0.9394 (left panel); cell: t(68) = 0.2249, *p* = 0.8227 (right panel); Figure 3F]. Interestingly, we observed a significant increase in the populational activity of VTA dopamine neurons reflected by the cell recorded/track, indicating the existence of compensatory effects on reduced neuronal activity [t(7) = 3.845, *p* = 0.0063; Figure 3G]. Taken together, these results suggest that Lkb1 is required for the maintenance of normal levels of burst activity in VTA DA neurons.

### Lkb1 deficiency in VTA DA neurons leads to impairments in social memory

**FIGURE 4:**
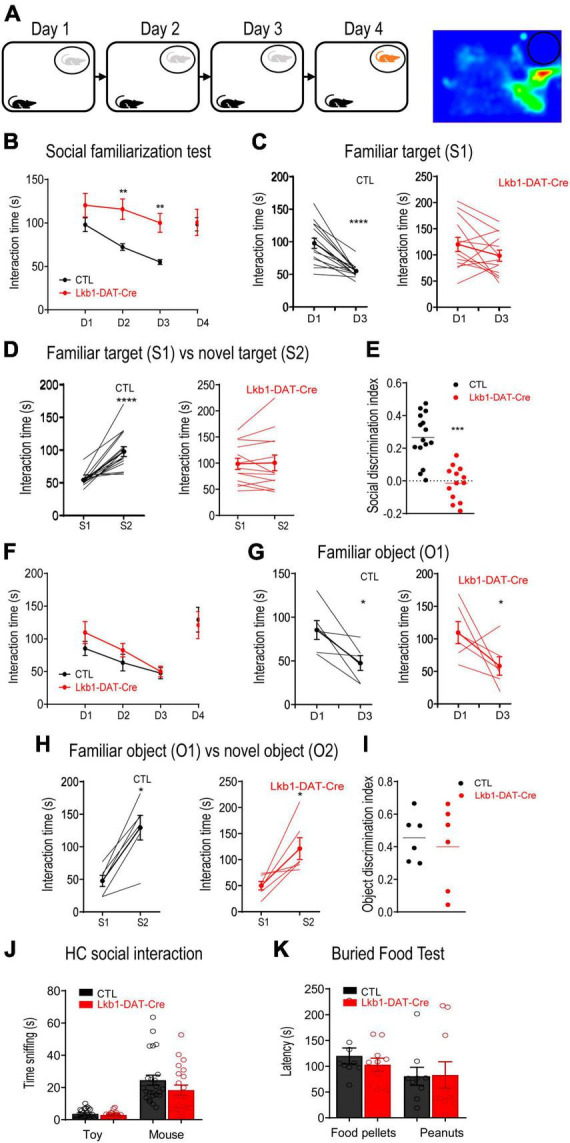
Liver kinase B1 (Lkb1)-DAT-Cre mice showed impairments in social memory. **(A)** Schematic diagram of the 3-day social familiarization test. **(B)** Social familiarization test. Lkb1-DAT-Cre mice spent significantly longer time interacting with the familiar social conspecifics (S1) throughout a 3-day familiarization test compared to the littermate control mice. **p < 0.01 compared with CTL. **(C)** Graph showing the interaction time at day 1 and day 3 with S1 in CTL and Lkb1-DAT-Cre mice. ****p < 0.0001 compared with D1. **(D)** Graph showing the interaction time at day 3 with S1 and at day 4 with S2 in CTL and Lkb1-DAT-Cre mice. ****p < 0.0001 compared with S1. **(E)** Social discrimination index calculated by (tS2-tS1)/(tS2+tS1). The social discrimination index of CTL mice was significantly higher than Lkb1-DAT-Cre mice. ***p < 0.001 compared with CTL. **(F)** Object familiarization test. Lkb1-DAT-Cre mice and CTL littermates spent a similar amount of time interacting with the Lego blocks (O1) during the 3-day object familiarization test. **(G)** Graph showing the interaction time at day 1 and day 3 with O1 in CTL and Lkb1-DAT-Cre mice. *p < 0.05 compared with D1. **(H)** Graph showing the interaction time at day 3 with O1 and at day 4 with O2 in CTL and Lkb1-DAT-Cre mice. *p < 0.05 compared with S1. **(I)** The object discrimination index calculated by (tO2-tO1)/(tO2+tO1). The object discrimination index was indifferent between CTL and Lkb1-DAT-Cre mice. **(J)** Home cage social interaction test. No significant difference was observed in time spent sniffing the social conspecific or toy when compared to Lkb1-DAT-Cre mice with littermate control mice. **(K)** Buried food test. The Lkb1-DAT-Cre mice showed similar latency in locating food pellets or peanuts in comparison with the littermate control mice. Data are presented with mean ± S.E.M.

The burst activity of DA neurons in the VTA mediates the role of dopamine in memory consolidation during reinforcement-guided learning (Clifton et al., 1992). The current findings showed that activation of VTA DA neurons was essential for the formation of social memory. Thus, we next examined whether Lkb1 was involved in social learning and memory. Lkb1-DAT-Cre and littermate CTL mice were subjected to a 3-day social familiarization test (Bariselli et al., 2018). The time the experimental mice spent initiating social interaction upon repeated exposure to a single social conspecific (S1) and the subsequent response to a novel social conspecific (S2) were assessed (Figure 4A). Repeated exposure to familiar social conspecifics resulted in a progressive reduction in interaction time in littermate CTL mice, leading to a significantly reduced time spent interacting with S1 on test day 3. However, Lkb1-DAT-Cre mice exhibited sustained longer interactions with S1 throughout the 3-day familiarization test [genotype: *F*_(1, 26)_ = 6.240, *p* = 0.0191; day: *F*_(3, 78)_ = 9.238, *p* < 0.0001; genotype × day: *F*_(3, 78)_ = 5.427, *p* = 0.0019; Figure 4B], resulting in no reduction in the time spent interacting with S1 [CTL: t(28) = 5.118, *p* < 0.0001; Lkb1-DAT-Cre: t(24) = 1.256, *p* = 0.2214; Figure 4C]. When tested for interaction with novel social conspecifics on subsequent test day 4, littermate CTL mice showed a significant increase in the total amount of time interacting with S2, whereas such an increase was not observed in Lkb1-DAT-Cre mice (CTL: t(28) = 5.357, *p* < 0.0001; Lkb1-DAT-Cre: t(24) = 0.1100, *p* = 0.9133; Figure 4D), therefore leading to a significantly lower social discrimination index (tS2-tS1)/(tS1+tS2) [t(26) = 5.727, *p* < 0.0001, Figure 4E].

To exclude the possibility that failed familiarization was due to impaired habituation, all mice were subjected to an object familiarization test. Mice of both genotypes showed a gradual decline in exploration of the same object upon repeated exposure [genotype: *F*_(1, 10)_ = 0.7260, *p* = 0.4141; day: *F*_(3, 30)_ = 11.47, *p* < 0.0001; genotype × day: *F*_(3, 30)_ = 0.5957, *p* = 0.6228; Figure 4F]. Both Lkb1-DAT-Cre and littermate CTL mice showed a significant decrease in the total amount of time spent interacting with O1 on day 3 [CTL: t_(10)_ = 2.744, *p* = 0.0207; Lkb1-DAT-Cre: t(10) = 2.307, *p* = 0.0437; Figure 4G] and a significant increase in time spent interacting with O2 on day 4 [CTL: t(10) = 3.935, *p* = 0.0028; Lkb1-DAT-Cre: t(10) = 3.169, *p* = 0.0100; Figure 4H]. Thus, there was no significant difference in the social discrimination index (tO2-tO1)/(tO1+tO2) [t(10) = 0.4585, *p* = 0.6564, Figure 4I], indicating intact habituation to the object in Lkb1-DAT-Cre mice. In addition, the significant increase in the total amount of time spent interacting with S1 observed in Lkb1-DAT-Cre mice was not due to hyperactivity during locomotion or to an increase in motivation to interact socially. This was evidenced by the lack of significant difference in the open-field test results [time: t(38) = 0.2981, *p* = 0.7673; distance: t(33) = 1.598, *p* = 0.1197, Supplementary Figure 2B] and in the home-cage social interaction test results [genotype: *F*_(1,43)_ = 2.067, *p* = 0.1578; target: *F*_(1,43)_ = 71.37, *p* < 0.0001; genotype × target: *F*_(1,43)_ = 1.623, *p* = 0.2095; Figure 4J], respectively, between the two genotypes.

Further, the buried food test results indicated normal olfactory perception in Lkb1-DAT-Cre mice [genotype: *F*_(1,17)_ = 0.1573, *p* = 0.6965; targets: *F*_(1,17)_ = 2.406, *p* = 0.1393; genotype × targets: *F*_(1,17)_ = 0.2528, *p* = 0.6216; Figure 4K]. These results suggest that Lkb1 expression in the VTA DA neurons is important for social memory formation. The function of VTA DA neurons in modulating anxiety-like behaviors has been well documented (Russo and Nestler, 2013; Berry et al., 2019; Morel et al., 2022). We also tested whether Lkb1 deficiency affected basal anxiety levels. In the open-field test, neither the time spent in the center nor the total distance traveled differed between Lkb1-DAT-Cre mice and littermate CTL mice [time: t(38) = 0.2981, *p* = 0.7673; distance: t(33) = 1.598, *p* = 0.1197; Supplementary Figure 2B]. There were also no genotype effects on the number of open arm entries [t(31) = 0.9336, *p* = 0.3577], number of total arm entries [t(31) = 0.6383, *p* = 0.5280], or time spent in the open arm [t(31) = 0.9495, *p* = 0.3497] in the elevated plus maze (Supplementary Figure 2C), indicating similar anxiety levels between Lkb1-DAT-Cre mice and littermate CTLs. These results support that the failure in familiarization observed in Lkb1-DAT-Cre mice was mainly due to the inability to form social memories of social conspecifics.

**FIGURE 5:**
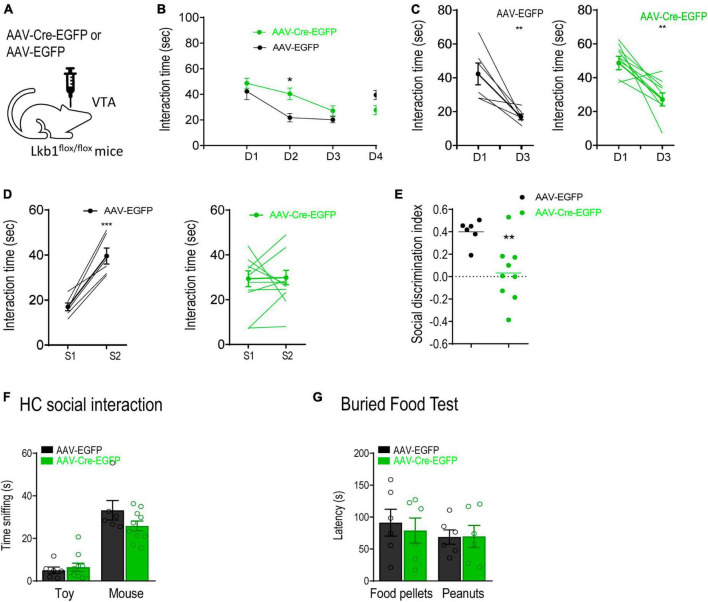
Liver kinase B1 (Lkb1) deletion mice showed impairments in social memory. **(A)** Schematic diagram showing virus-mediated deletion of Lkb1 in the VTA of adult mice. **(B)** AAV-Cre-EGFP mice spent significantly longer time interacting with the familiar social conspecifics (S1) throughout a 3-day familiarization test compared to the AAV-EGFP mice. *p < 0.05 compared with AAV-EGFP. **(C)** Graph showing the interaction time at day 1 and day 3 with S1 in AAV-EGFP and AAV-Cre-EGFP mice. **p < 0.01 compared with D1. **(D)** Graph showing the interaction time at day 3 with S1 and at day 4 with S2 in AAV-EGFP and AAV-Cre-EGFP mice. ***p < 0.001 compared with S1. **(E)** The social discrimination index of AAV-EGFP mice was significantly higher than AAV-Cre-EGFP mice. **p < 0.01 compared with AAV-EGFP. **(F)** Home cage social interaction test. No significant difference was observed in time spent sniffing the social conspecific or toy when comparing AAV-Cre-EGFP mice with AAV-EGFP mice. **(G)** Buried food test. The AAV-Cre-EGFP mice showed similar latency in locating food pellets or peanuts in comparison with the AAV-EGFP mice. Data are presented with mean ± S.E.M.

Given that Lkb1 is crucial for neuronal development, we next investigated whether the impairment in social memory in Lkb1-DAT-Cre mice was due to alterations in VTA DA neurons during development. The total number of DA neurons immune-stained for TH in the VTA of adult Lkb1-DAT-Cre mice and littermate CTLs was not significantly different [t(4) = 0.1656, *p* = 0.8765; Supplementary Figure 3], suggesting that Lkb1 deficiency did not alter the number of DA neurons in the VTA. To further confirm whether Lkb1 played a role in modulating the function of post-developmental DA neurons in the VTA and related behaviors, we used AAV-Cre-EGFP to delete Lkb1 from the VTA of adult mice. The Lkb1^fl/fl^ mice received bilateral intra-VTA injections of AAV-Cre-EGFP or AAV-EGFP (Figure 5A). The Cre expression was selective for dopaminergic neurons, suggesting confined Lkb1 loss (Supplementary Figure 4A). Similar to our findings in conditional knockout mice, mice with a Lkb1 deletion in the VTA exhibited a slower rate of familiarization during repeated exposure to the same social conspecifics [genotype: *F*_(1, 14)_ = 1.468, *p* = 0.2456; day: *F*_(3, 42)_ = 12.13, *p* < 0.0001; genotype × day: *F*_(3, 42)_ = 5.826, *p* = 0.0020; Figure 5B]. These mice spent significantly more time interacting with the familiar social conspecific on day 2 (*p* = 0.0143). Further, when analyzing the time spent interacting with S1 throughout the 3-day familiarization session, the mice behaved similarly to mice injected with the control virus [AAV-EGFP: t(10) = 3.809, *p* = 0.0034; AAV-Cre-EGFP: t(18) = 3.909, *p* = 0.001. Figure 5C].

Interestingly, in the social discrimination test, mice in the AAV-Cre-EGFP group showed no preference for the novel conspecific [AAV-Cre-EGFP: t(16) = 0.1148, *p* = 0.9100; AAV-EGFP: t(10) = 5.658, *p* = 0.0002; Figure 5D], leading to a reduced social discrimination index in mice microinjected with AAV-Cre-EGFP in the VTA [t(13) = 2.438, *p* = 0.0299; Figure 5E]. The home-cage interaction test confirmed that sociability between the two groups of mice was similar [group: *F*_(1,14)_ = 1.584, *p* = 0.2288; target: *F*_(1,14)_ = 64.79, *p* < 0.0001; group × target: *F*_(1,14)_ = 2.193, *p* = 0.1608; Figure 5F], suggesting that no hypersociability occurred following Lkb1 conditional knockout in the VTA muscle. In addition, mice in the AAV-Cre-EGFP group showed normal olfactory perception as assessed using the buried food test [group: *F*_(1,10)_ = 0.06395, *p* = 0.8055; target: *F*_(1,10)_ = 2.496, *p* = 0.1452; group × target: *F*_(1,10)_ = 0.4377, *p* = 0.5232; Figure 5G]. This result excluded the possibility that deficits in familiarization were induced by olfactory function. We also tested the mice for anxiety-like behaviors and observed no difference in either the open-field test [t(13) = 1.093, *p* = 0.2944; Supplementary Figure 4B] or the elevated plus maze test [t_(12)_ = 1.679, *p* = 0.1190; Supplementary Figure 4C] findings. These results highlight the behavioral phenotype of Lkb1-DAT-Cre mice, showing a slower rate of familiarization. In combination with our findings in conditional knockout mice, these results confirm that Lkb1 plays an essential role in social memory processing.

## Discussion

Several factors modulate the activity of dopaminergic neurons in the VTA. Here, we show that the activation of VTA dopaminergic neurons is required for social memory formation. The inhibition of VTA dopaminergic neurons impairs the formation of social memory, as evidenced by sustained social interaction and a lack of preference for social novelty. In addition, we find that the serine/threonine protein kinase Lkb1 is required to maintain the activity state of dopaminergic neurons in the VTA. Genetic deletion of Lkb1 in the VTA dopaminergic neurons reduces the frequency of burst firing without affecting tonic firing rate. Furthermore, Lkb1 deletion during the developmental stage of dopaminergic and mature neurons in the VTA leads to impairment in social memory formation. To the best of our knowledge, these findings provide the first direct evidence that Lkb1 plays an important role in the modulation of dopaminergic neuronal activity and social behavior.

The mesolimbic dopamine system, which originates from VTA dopaminergic neurons, is well characterized for its regulation of executive, affective, and motivational functions (Serafini et al., 2020). Recent studies have provided direct evidence that the VTA dopaminergic system is activated during social interactions, and is closely involved in the regulation of social behaviors, including social interaction, avoidance, novelty, and social learning (Krishnan et al., 2007; Gunaydin et al., 2014; Bariselli et al., 2018; Solie et al., 2022). Recognizing and memorizing familiar vs. novel social conspecific is required for an individual to express appropriate behavioral responses and is the foundation for establishing meaningful social relationships (van der Kooij and Sandi, 2012). Social memory, a key component of social cognition, is formed and stored in the hippocampus (Hitti and Siegelbaum, 2014; Okuyama et al., 2016; Meira et al., 2018), mainly in the CA2 and CA1 subregions. The DA system modulates hippocampus-dependent learning and memory (Lisman and Grace, 2005; Lisman et al., 2011; Broussard et al., 2016). Therefore, we tested whether dopaminergic neurons in the VTA were directly involved in modulating social memory. Our results showed that the number of c-fos-positive cells in VTA DA neurons was consistently higher during social interaction, as well as 6 h after social interaction, in mice that interacted with social conspecifics than in mice that interacted with an object. This finding suggested that VTA dopaminergic neurons could participate in modulating social memory. Using hM4Di-DREADD-mediated control of neuronal activity, we showed that inhibition of VTA DA neurons before social interaction significantly impaired social discrimination 24 h later, confirming that dopaminergic neurons in the VTA was required for the formation of social memory. The lack of social preference induced by DA neuron inhibition before the social interaction test was not due to the inability to recognize novel conspecifics, impaired sociability or olfactory function accessed by objection recognition test, home-cage sociability test and buried food test, respectively.

Social memory is stored in the hippocampus (Hitti and Siegelbaum, 2014; Okuyama et al., 2016) which receives relatively less intense but direct projections from the VTA dopaminergic system (Gasbarri et al., 1994b; Tsetsenis et al., 2021). The release of dopamine in the hippocampal formation enhances long-term potentiation induction in CA1, thus facilitates learning (Lisman and Grace, 2005). Interestingly, novelty itself can induce dopamine release in the hippocampus from both the VTA and the locus coeruleus. The former is responsible for the enhancement of memory retention that requires the reactivation of the existing memory (McNamara et al., 2014; Tsetsenis et al., 2021), while the latter is engaged in memory consolidation in response to infrequently occurring events (Kempadoo et al., 2016; Takeuchi et al., 2016; Duszkiewicz et al., 2019). This strongly suggests the distinct contribution of the two dopamine-releasing systems to novelty-induced memory retention. For example, in aversive memory formation, VTA-hippocampal projections are sufficient to promote memory formation (Tsetsenis et al., 2021). Considering the close involvement of VTA DA neurons in modulating social behavior, we speculated that the VTA-hippocampal route could play an important role in social memory consolidation. A recent study further supports this speculation by showing that the activity of VTA DA neurons encodes a social prediction error that drives social reinforcement learning (Solie et al., 2022). However, further research is needed to directly elucidate the specific roles of hippocampal-projecting VTA DA neurons and locus coeruleus catecholamine neurons in the retention of memory of interaction with a novel social conspecific. In addition to memory consolidation, VTA DA neurons have also been reported to enhance memory retrieval (Clos et al., 2019); however, our results showed that inhibition of DA neurons before the retrieval of social memory did not affect social discrimination. This suggested that VTA DA neurons might not be involved in the retrieval of social memory. Social memory is formed and stored in the CA1 subregion of the hippocampus (Okuyama et al., 2016), which, among all hippocampal subregions, predominantly receives dopaminergic projections from the VTA (Adeniyi et al., 2020). Although the ventral CA1 is also required for social memory retrieval, the ventral CA1 participates in social memory retrieval as a result of its input from the dorsal CA2 (Meira et al., 2018). The dorsal CA2 receives relatively less dopaminergic innervation, and this could be the underlying mechanism for the lack of regulation of VTA DA neurons in social memory retrieval.

The activity of the dopaminergic neurons in the VTA is regulated by both intrinsic properties and synaptic transmission (Gantz et al., 2018). Lkb1, a ubiquitously expressed master kinase, plays a crucial role in regulating numerous cellular events via the activation of AMP-activated protein kinase subfamily members (Shackelford and Shaw, 2009). Despite its diverse functions in the peripheral system, the roles of Lkb1 in the developing and mature nervous system, such as neuronal migration (Asada et al., 2007), neurite outgrowth (Huang et al., 2014), maintenance of neural integrity (Barnes et al., 2007), stabilization of intrinsic neuronal excitability (Levitan et al., 2020), and modulation of pre- or post-synaptic responses (Claret et al., 2011; Kwon et al., 2016), also indicate that it is a multifunctional master kinase in the CNS. Lkb1 is ubiquitously expressed in the brain, including the midbrain (Barnes et al., 2007; Shelly et al., 2007). We provide direct evidence that Lkb1 is required for maintaining the phasic firing activity of dopaminergic neurons in the VTA by showing that the conditional deletion of Lkb1 in VTA DA neurons significantly reduces the frequency of burst firing of these neurons. Notably, we observed such deficits in the mice with genetic deletion of Lkb1 in the VTA DA neurons although we did not observe changes in numbers of DA neurons, suggesting that expression of Lkb1 during the developmental stage is required for functional maturation of DA neurons. The tonic activity of the VTA DA neurons did not change significantly after Lkb1 deletion. Interestingly, we noted a clear non-normal distribution of data obtained in the conditional KO mice, with over half of the total neurons recorded displaying very low burst activities and the others showing neuronal activities comparable to those of the control mice. This implied that the Lkb1 protein was possibly expressed in only a subpopulation of VTA DA neurons or it was expressed in all VTA DA neurons, but some co-express another gene with redundant function. When further separated the recorded neurons using location of the cells, a relatively much more noticeable reduction of burst firing was identified in the neurons located to the ventral-medial part of VTA (data not shown), further indicating that Lkb1 deletion exerted differential modulatory effects on VTA DA neurons. Further studies are needed to confirm the neurochemical and projection properties of these neurons.

The bursting activity of DA neurons in the VTA relies on glutamate input (Grace et al., 2007). Lkb1 has been shown to directly and indirectly modulate glutamate receptor signaling, and our current findings support the notion. Kone et al. (2014) reported that the transcriptional levels of several genes associated with AMPA receptor functions, such as gria1, gria3, Nptx2, and dlgap2, were significantly altered with Lkb1 deletion in pancreatic β cells (Kone et al., 2014). Furthermore, several molecules upstream and downstream of Lkb1 have been reported to coordinate glutamate receptor signaling. Lkb1 acts as an upstream kinase mediating the NMDA receptor-dependent activation of Par1 during activity-dependent synaptic remodeling (Bernard and Zhang, 2015). The essential components of Lkb1 signaling, such as APPL1 and AMP-activated protein kinase, have also been reported to coordinate glutamate receptor signaling (Ahmadi and Roy, 2016; Hua et al., 2023). These studies strongly suggest that Lkb1 in VTA DA neurons dampens bursting activity by affecting postsynaptic glutamate transmission. Although we did not observe significant changes in the total number of VTA DA neurons in Lkb1 conditional knockout mice, we could not rule out the possibility that the changes in the electrophysiological properties of VTA DA neurons were due to structural abnormalities. This was because Lkb1 played a crucial role in mediating various steps in neural development (Asada et al., 2007; Courchet et al., 2013; Huang et al., 2014).

Given that DA neuronal activity was required for social memory formation and that Lkb1 was essential for maintaining the bursting activity of VTA DA neurons, we further tested the behavioral phenotypes of mice with Lkb1 deficiency in VTA DA neurons to confirm the role of VTA DA neurons in social cognition. Consistent with the electrophysiological results, the data showed that, compared with CTL mice, mice with either genetic deletion of Lkb1 in VTA DA neurons or viral-mediated conditional knockout of Lkb1 in the VTA during adulthood spent significantly longer time interacting with familiar social conspecifics during a 3-day social familiarization test. The motivation to initiate social interactions with novel social conspecifics was not affected. Owing to the increase in time spent interacting with familiar social conspecifics, the social discrimination index in mice lacking Lkb1 in VTA DA neurons was significantly impaired. The home-cage interaction test for assessing sociability further confirmed that Lkb1 in VTA DA neurons did not impair the motivation to interact with social conspecifics. Interestingly, mice with a virus-mediated conditional knockout of Lkb1 in the adult VTA partially recapitulated the behavioral phenotypes observed in mice with conditional Lkb1 deletion. The virus-mediated conditional knockout of Lkb1 was slower than the impaired familiarization with S1, possibly due to the failure of social memory formation. These mice also displayed impaired social novelty preference even when they were completely familiar with S1. Given that we could not rule out the possibility that virus-mediated conditional knockout of Lkb1 could occur in other cell types in the VTA of adult mice, these results prompted us to further dissect the circuitry basis and diverse functions of Lkb1 in the VTA. Along with the results obtained using *ex vivo* electrophysiological recordings, these results strongly suggest that the effects of Lkb1 on social memory formation occur mainly through the functional modulation of DA neurons.

In summary, our results provide direct evidence that the activation of VTA DA neurons is required for social memory formation. In addition, to our best knowledge, we are the first to report the functional role of Lkb1 in modulating the activity of VTA DA neurons and its crucial role in regulating social behavior. The current findings reveal new cellular and molecular mechanisms underlying social memory processing and thus provide instrumental evidence for the development of novel therapeutic strategies for psychiatric disorders associated with social impairment.

## Data availability statement

The original contributions presented in this study are included in the article/Supplementary material, further inquiries can be directed to the corresponding authors.

## Ethics statement

The animal study was approved by the Institutional Animal Care and Use Committees of Qilu Hospital of Shandong University. The study was conducted in accordance with the local legislation and institutional requirements.

## Author contributions

DZ: Conceptualization, Formal analysis, Funding acquisition, Resources, Supervision, Validation, Writing – review and editing. MY: Data curation, Investigation, Methodology, Writing – original draft. BH: Supervision, Project administration, Writing – review and editing. FS: Investigation, Writing – review and editing. GX: Validation, Writing – review and editing. YZ: Investigation, Writing – review and editing. XW: Formal analysis, Writing – review and editing. XiL: Formal analysis, Investigation, Writing – review and editing. XgL: Funding acquisition, Supervision, Writing – review and editing.
