# Supplementary figures and images for: Liver kinase B-1 modulates the activity of dopamine neurons in the ventral tegmental area and regulates social memory formation
Source: Front Mol Neurosci. 2024 Apr 5;17:1289476. doi: 10.3389/fnmol.2024.1289476 (PMC11026561; doi:10.3389/fnmol.2024.1289476)

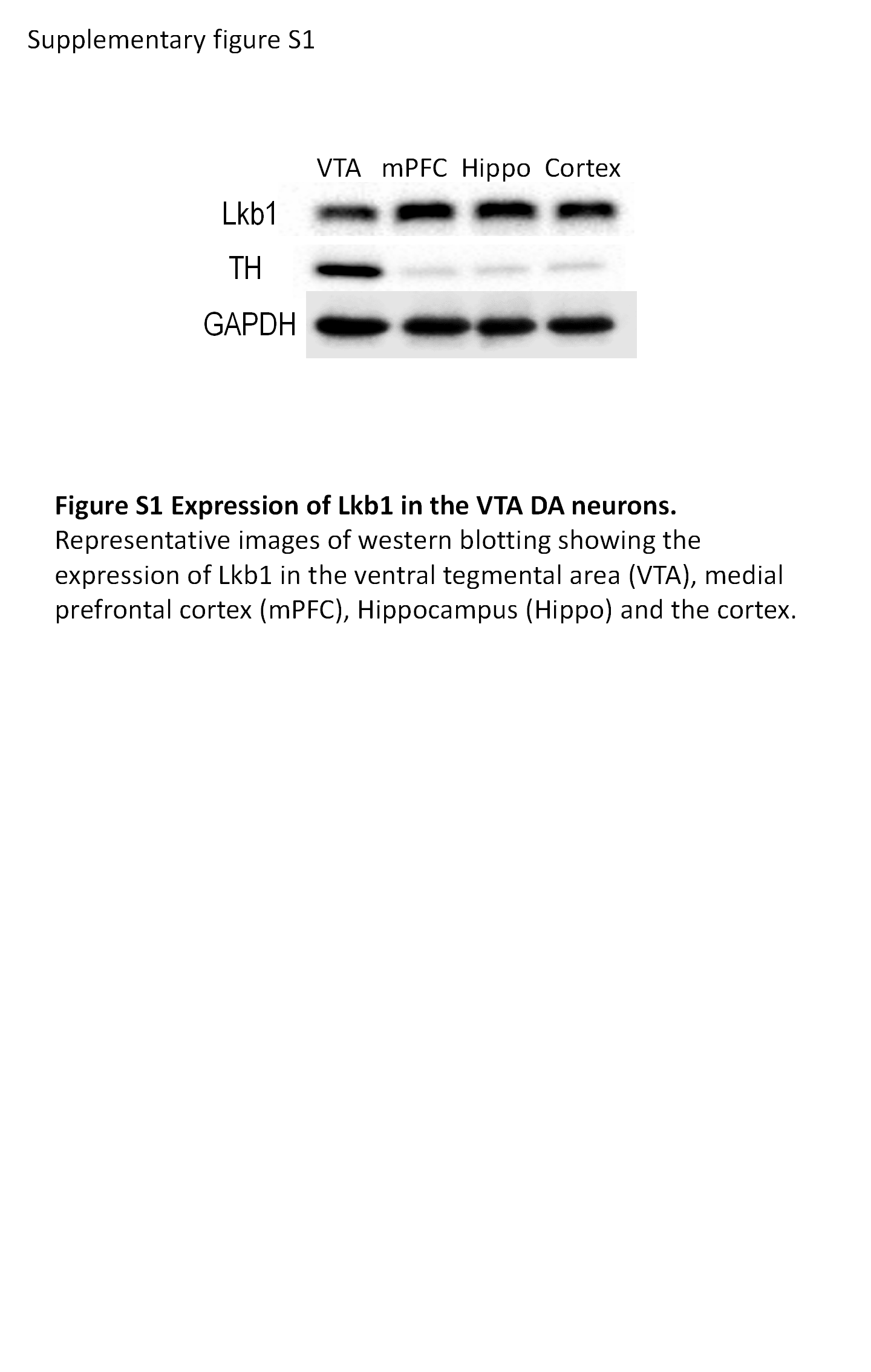

Supplement: Supplementary file 1 [file Image_1.tif]

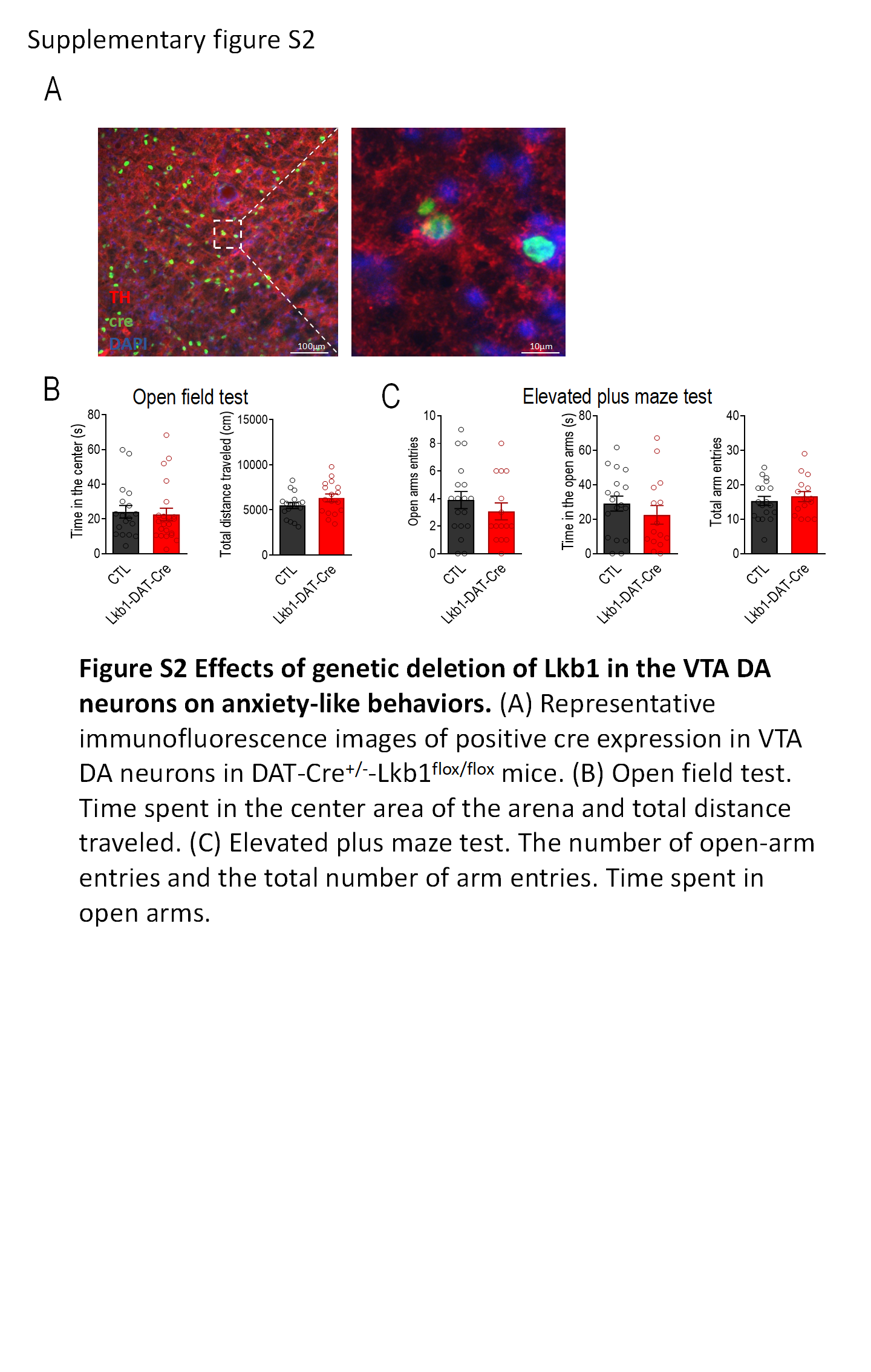

Supplement: Supplementary file 2 [file Image_2.tif]

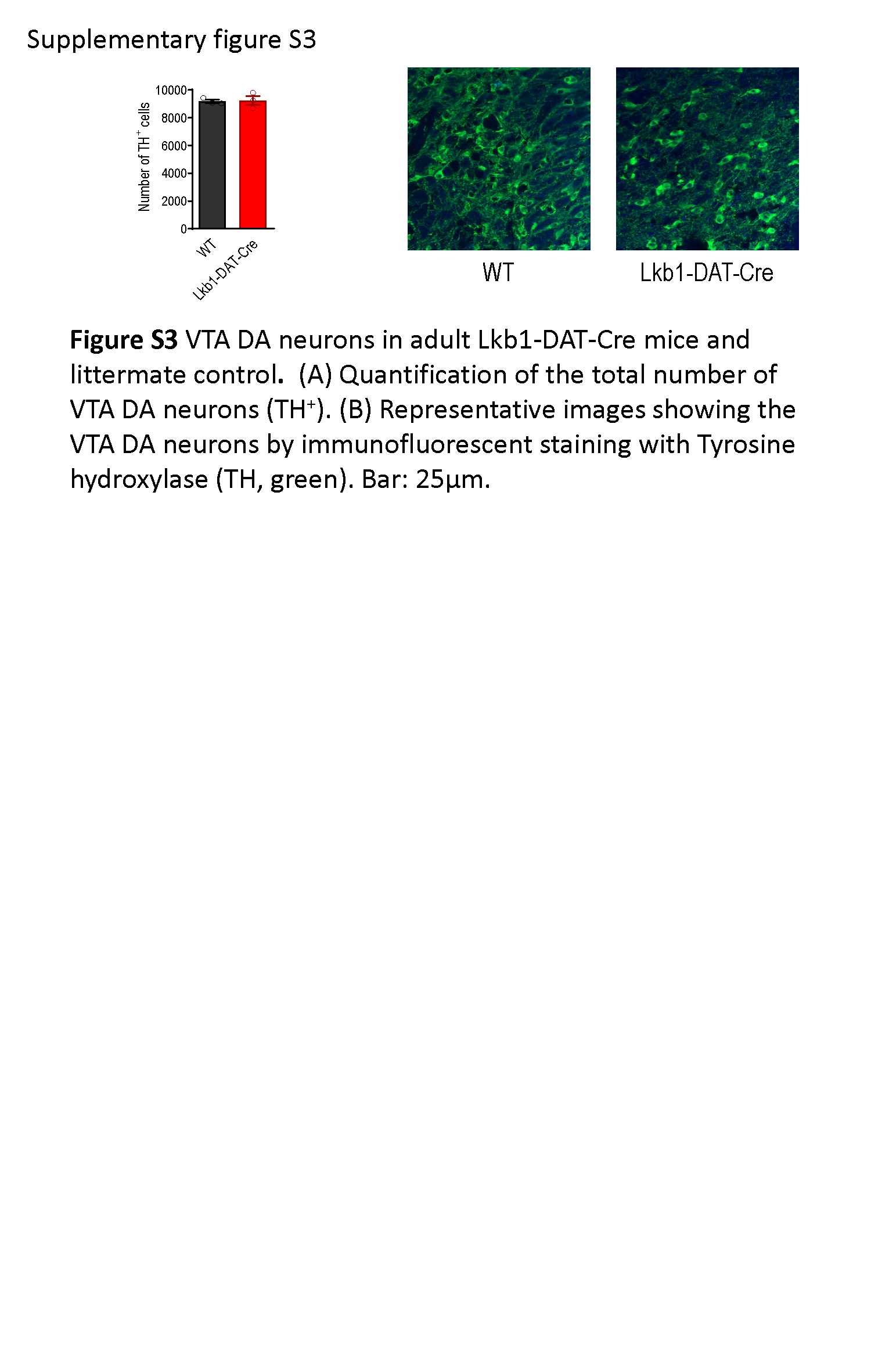

Supplement: Supplementary file 3 [file Image_3.TIF]

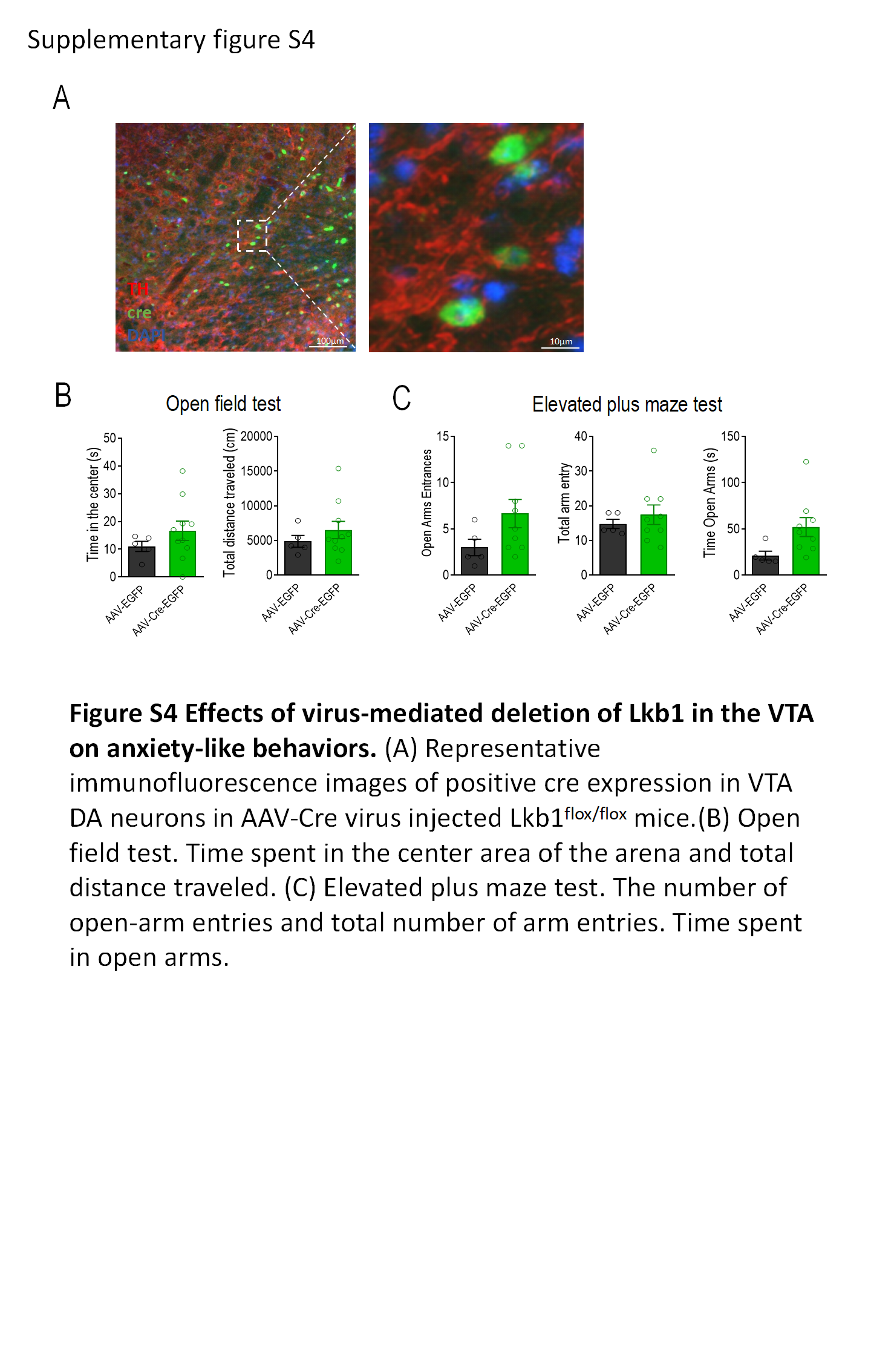

Supplement: Supplementary file 4 [file Image_4.tif]
